# Complications of the “Nuss Procedure” In Pectus Excavatum

**Published:** 2020-05-31

**Authors:** A. Garzi, M. Prestipino, M.S. Rubino, R.M. Di Crescenzo, E. Calabrò

**Affiliations:** 1 Division of Pediatric M.I.S. and Robotic Surgery University of Salerno, Italy; 2 Division of Pediatric Surgery A.O. S. Maria della Misericordia Perugia, Italy; 3 Department of Advanced Biomedical Sciences, Pathology Unit, University of Naples Federico II

**Keywords:** pectus excavatum, pediatrics, pediatric complications, Nuss procedure

## Abstract

During the pediatric age range, one the most frequent deformities of the chest wall are Pectus Excavatum (PE). Currently the treatment of choice for PE is surgical intervention following the Nuss procedure.

In this study, we present a description of the complications associated with surgical treatment of PE with the Nuss technique, in patients with symmetrical or asymmetrical deformity, in different stages of disease severity.

The study was conducted in collaboration with the Pediatric Hospital “Istituto G. Gaslini” of Genoa. We analyzed a cohort of 402 patients (334 males and 68 females), who underwent corrective surgery between 2005 and 2018. Within this group of patients, we observed 82 cases with complications (20.39%), 20 of which were intraoperative (4.98%) and 62 post-operative (15.42%). For the evaluation of complications, this group was arbitrarily divided into patients with symmetric and asymmetric Pectus and in patients with mild, moderate and severe Pectus using Haller’s index.

Although a small group of patients presented complications, overall results from data analysis show that the Nuss technique represents the preferred surgical procedure for the treatment of PE, in agreement with literature. Furthermore, in our results show no correlation between asymmetry or severity of PE with complication related to the surgery.

## I. INTRODUCTION

PE is a congenital deformity of the anterior chest wall and sternum, which is believed to be a result of abnormal growth of the ribs and cartilage. PE occurs in about 1 out of every 300 to 400 live births. Male patients with pectus excavatum outnumber female patients at a ratio of 2:1 to 9:1 and the vast majority of patients are of Caucasian descent (1). The condition is uncommon among African Americans and Latinos (2).

The clinical workup of a patient with PE requires an accurate assessment of the severity of the defect to identify patient suitable for surgery [2]. In mild cases, medical treatment is the first-line approach; in severe PE or in patients in which medical treatment has failed, surgery is the best therapeutic choice.

Nowadays, the best treatment for PE is surgery with Minimally invasive repair of pectus excavatum (MIRPE), also known as the “Nuss procedure”, which has gained considerable popularity since its introduction in 1998. This technique is widely spread and, in many countries, is considered as the standard of care for the surgical correction of PE. It’s important to underline that this procedure is safe and effective [1] when performed in a center where surgeons have experience in this kind of disease [3, 4].

The aim of this study is to evaluate the safety of Nuss technique for surgical treatment of patients with symmetrical or asymmetrical PE, in different stages of disease.

## II. MATERIALS AND METHODS

The studied population included pediatric patients with PE, who underwent surgical treatment with the Nuss technique at the Pediatric Surgery Divisions of the Pediatric Hospital “Istituto G. Gaslini” of Genoa and of the University Hospital of Salerno. Before surgery, clinical features and patient’s’ comorbidities were assessed. Comorbidities were subdivided into four groups: cardiac disorders, lung disorders, connective tissue disorders and other congenital anomalies / genetic syndromes.

We performed a descriptive analysis, which included demographic ad clinical baseline variables, as well as data on intra- and post-operative patients’ outcome.

Categorical variables were expressed as number and percentage.

Complications were compared in patient groups with Symmetric and Asymmetric Pectus and in patient groups with Mild, Moderate and Severe PE. This data was assessed by using the χ2 test.

The patients were followed up at 1, 6, 12, and 24 months after surgery.

### Notes on surgical technique

The technique used is the following: the first step is to determine the length of the bar to be used for the repair. This can be achieved by measuring the distance between the right and left midaxillary lines. Most surgeons prefer to use a bar shorter than one or two inches.

Bilateral transverse incisions are made between the anterior and posterior axillary lines on each side of the chest, at the level of the deepest part of the defect [5]. Next, a thoracoscope is inserted into the left pleural cavity through a left surgical access on the anterior axillary line [6].The trocar in the right chest is placed on the anterior axilla, several ribs below the level where the bar will be inserted. The trocar for the left thorax is positioned in the space between the second and third ribs on the anterior axillary line avoiding the heart, which in these patients is shifted to the left due to the malformation [7]. A dissector of adequate size is chosen and is inserted into the thorax through the surgical access on the right side. By means of rotational and forward movements it is carried through the mediastinum (with the tip facing forward) [5]. This creates the tunnel where the metal bar will be accommodated.

The previously selected metal corrective bar is introduced into the chest [6]. 1, 2 or 3 bars can be inserted.

Once the bar is inserted, it must be rotated using two tools (flippers) which allow the synchronized 180 ° rotation of the bar until it assumes the final convex position such as to push the sternum out and correct the sternal deformity.

Next, the bar is fixed. Currently, absorbable sutures and unilateral or bilateral stabilizers on the bar are used. [5]. After implantation of the bar, the correct position of the implants is evaluated in bilateral thoracoscopy, as well as the retrosternal space, the sternum, the pericardium, the lung and the diaphragm to exclude any injuries.

Pneumothorax is evacuated.

A thoracic drainage tube is inserted into the right pleural space and is maintained for the next 2 days [6].

A postoperative radiograph is usually performed in surgery to confirm bilateral lung expansion and evaluate the position of the bar [6].

The bars are kept in place, on average, for 3 years. Then, the bars are removed through an incision made along the previous scar [5].

## III. RESULTS

In this study we analyzed a total of 402 patients, of which 334 males (83%) and 68 females (17%), who underwent surgery between 2005 and 2018. The average age at repair of PE was 14.95 years for males and 13.44 years for females. The most commonly observed comorbidities associated with PE were heart disease, lung disease and connective tissue disorder (Table 1).

In relation to symmetry, most of the patients had an asymmetric Pectus (67.66; N= 272), while 32.34% (N = 130) had symmetric Pectus.

With regard to the severity of the disease, patients were divided into mild (N = 24), moderate (N = 35) and severe (N = 343), using the Haller’s index, a severity index calculated by CT evaluation that derives from the division of the anterior-posterior diameter by the transverse diameter. Normal measurement is approximately 2.5. An index greater than 3.2 has been correlated with severe deformity that often requires surgery. The average Haller’s index was 5.2 (range 1.67–22).

For all the procedures the average surgical time of 85 minutes. The mean patient hospitalization was 6 days.

The total number of complications for bar placement was 20.40% (N = 82), of which 4.98% (N = 20) intraoperative and 15.45% (N = 62) postoperative and there was no intra and postoperative mortality.

The major problems were the intraoperative pericardial openings (N = 10, 2.49%) and the dislocation of the bar during the postoperative period(N= 16; 3.98%), which required a re-intervention for repositioning.

Table 2 describes all intra- and post-operative complications.

Complications were compared in patient groups with Symmetric and Asymmetric Pectus and in patient groups with mild, moderate and severe PE (table 3 and Table 4).

### TEST χ2 FOR SYMMETRIC / ASYMMETRIC PE

Significance level: 0.05

**Results:**

The χ2 is 0.3004.

The p-value is 58364.

Using the Test χ2, the result is not statistically significant for p <0.05.

### TEST χ2 FOR MILD, MODERATE AND SEVERE PE

Significance level: 0.05

**Results:**

The χ2 is 4.9727.

The p-value is 083214.

Using the Test χ2, the result is not statistically significant for p <0.05.

The comparison between patient with symmetric and asymmetric PE and patients with mild, moderate and severe PE showed that there are no differences in the various groups regarding the complications of MIRPLE. Therefore, the gravity and symmetry of the PE do not affect the onset of complications.

## IV. DISCUSSION

The aesthetic effects after corrective rib cage surgery using the Nuss procedure are quite satisfactory. According to various Authors, surgery is successfully performed in over 90% of cases [8, 9, 10]. The results are appreciable, with a low complication rate and minimal scarring [11, 12].

Morbidity is usually transient and reversible, although convalescence may be long-lasting.

The percentage of complications and failures found in this study was 20.40%; which is comparable to those listed in other studies performed in groups of patients who have more than 200 subjects of variable age, such as 16.3% [13], 18.7% [3], or 35.0% [14].

Is important to underline that the frequency of complications decreases with the acquisition of surgical experience. No differences in the incidence of complications were found neither between symmetric and asymmetric Pectus nor in patients in different stages of disease, defined by the Haller index.

Analyzing the complications, one of the most common, which requires surgical revision after MIRPE, was the displacement of the bar. The rate of this complication, reported in several studies at the beginning of the experience with this procedure, was greater than 10% [15, 16–18]. This percentage drastically decreased with the introduction of lateral stabilizers and/or additional fixation points [16]. We observed a the bar displacement frequency of in 3.98% of cases and it is, however, necessary to carry out an extended follow-up to establish the accurate incidence of this complication.

## V. CONCLUSION

The “Nuss procedure” is certainly the currently preferred technique for the treatment of PE, with an appreciable success rate and a short hospitalization. Our results are consistent with previous studies, confirming the good safety and effectiveness profile of the procedure in pediatric age. In addition, these results confirm that the severity and symmetry of the pectus do not influence the surgical risk.

## Figures and Tables

**Table 1:** Clinical and demographic characteristics

	Clinical and demographic characteristics
**Patients**	N = 402
**Age (years)**	M→ 14,95 years old(range 4,9–27,64) F→ 13,44 years old(range 5,71–26.73)
**Sex, N (%)**	Male 334 (83,08%) Females 68 (17%)
**Simmetry, N (%)**	→M 110 (27,36%) 20 (4,97%)→F
**Asimmetry, N (%)**	224 (55,72%)→M 48 (11,94%)→F
**Comorbidities**	Flaring ribs →11(2.74%) Breast asymmetry → 4 (0.99%) Cardiac dislocation → 9 (2.24%) Mitral valve prolapse → 3 (0.75%) right incomplete bundle branch block → 5 (1.24%) Delay of right intraventricular conduction → 1 (0.25%) High blood pressure → 1 (0.25%) Poland syndrome → 1 (0.25%) Noonan syndrome → 2 (0.50%) suspected Marfan Syndrome →1 (0.25%) Sternal cleft → 1 (0.25%) Diaphragmatic eventratio → 1 (0.25%) Excavatum-carenatum → 1 (0.25%) Nephrotic syndrome → 1 (0.25%) Megalencephaly → 1 (0.25%) Lung disease → 2 (0.50%) Relapse in patients already operated with Nuss technique → 2 (0.50%) Previous cardiac surgery with development of sternal adhesions → 1(0.25%)

**Table 2:** Complications: intra and post-operative

Complications	Intraoperative	Postoperative
**Pericardium Opening**	10 (2,49%)	
**Pneumothorax**	5 (1,24%)	7 (1,74%)
**Hemothorax**	2 (0,50%)	4 (1,00%)
**Arrhythmias**	2 (0,50%)	
**Serious Hypotension on waking**	1 (0,25%)	
**Bar Displacement**		16 (3,98%)
**Pleural Effusion**		14 (3,48%)
**Wound Infections**		9 (2,24%)
**Pericardial Effusion**		5 (1,24%)
**Lung Thickenings**		5 (1,24%)
**Minor Neurological Deficits**		2 (0,50%)

**Table 3:** Symmetric and Asymmetric PE

	Complications N=71 (17.66%)	No complications N=331(82.34%)	Total N=402(100%)
**PE SYMMETRIC**	21 (16.15%)	109 (83.85%)	130 (100%)
**PE ASYMMETRIC**	50 (18.38%)	222 (81.62%)	272(100%)

**Table 4:** Mild, moderate and severe PE

	Complications N=81(20.15%)	No Complications N=321(79.85%)	Total N=402 (100%)
**PE MILD**	5 (20.83%)	19 (79.17%)	24 (100%)
**PE MODERAT E**	2 (5.72%)	33 (94.28%)	35 (100%)
**PE SEVERE**	74 (21.57%)	269 (78.43%)	343 (100%)
